# Two-Stage Arthrodesis after an Infected Total Knee Replacement Using a Coupled Nail and Dual-Plate Construct: A Third-World Solution and Review of Options

**DOI:** 10.1155/2020/1762369

**Published:** 2020-02-10

**Authors:** Marlon M. Mencia, Raakesh Goalan, Atiba Akii Bua

**Affiliations:** ^1^Port of Spain General Hospital, Port of Spain, Trinidad and Tobago; ^2^Eric Williams Medical Sciences Complex, St. Joseph, Trinidad and Tobago

## Abstract

Infection following total knee arthroplasty is a serious and increasingly common complication. Several treatment options are available. Although a two-stage revision remains the gold standard, salvage procedures are sometimes needed. We describe a case of an infected knee arthroplasty that was salvaged using a novel technique combining two linked intramedullary nails and bilateral compression plating.

## 1. Introduction

Total knee arthroplasties (TKAs) are increasing in frequency and are easily the most commonly performed joint replacement procedure worldwide [1]. Infection is arguably the most devastating complication with a reported incidence from 1.1% to 12.4% [2].

The gold standard for the treatment of periprosthetic joint infection (PJI) is a two-stage revision arthroplasty, with excellent success rates for the eradication of infection [3].

When a two-stage revision fails or is not feasible, a salvage procedure is warranted to retain functionality of the involved limb. Of the salvage procedures, arthrodesis provides the best postoperative function, yet the most effective technique for arthrodesis in the setting of infection remains controversial [4–6].

We describe a case of periprosthetic infection following a primary knee replacement which was treated with a novel technique using a combination of two linked intramedullary nails and bilateral compression plating.

## 2. Case Presentation

**Figure 1:**
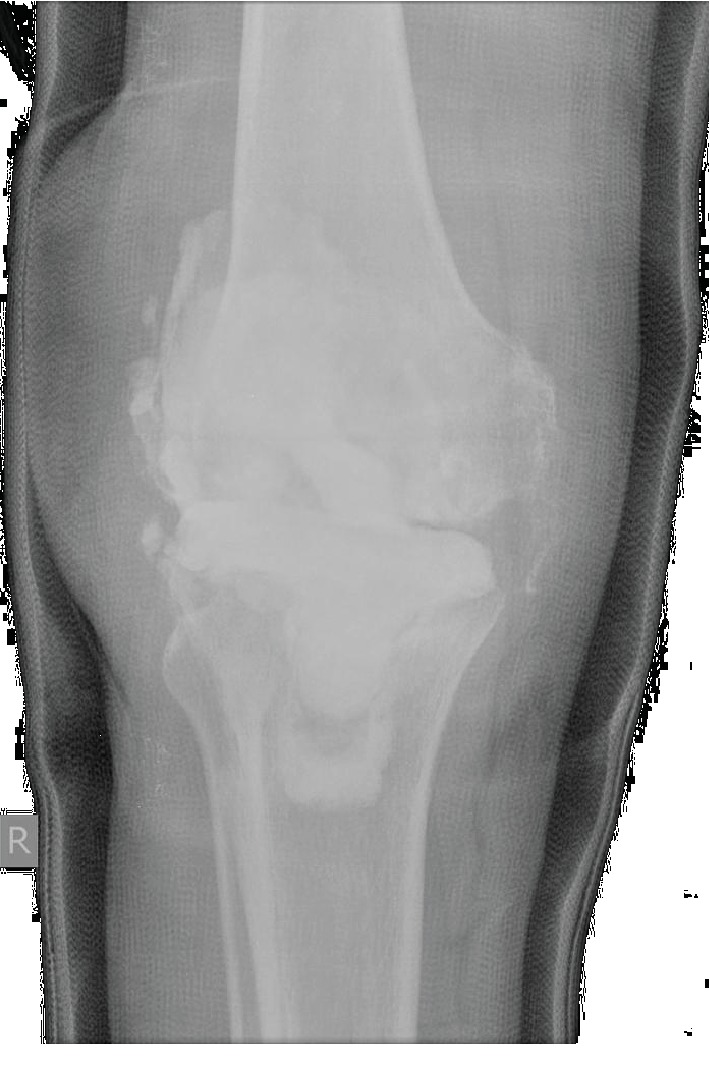
Radiograph shows cement spacer in situ.

A 62-year-old male, with a chronic draining sinus over the lateral aspect of his knee presented to the orthopaedic clinic 2 years following a right total knee replacement. His postoperative course was complicated by an early deep periprosthetic infection which was initially treated with irrigation and debridement but eventually required removal of the implants and insertion of an articulating antibiotic-impregnated cement spacer Figure 1.

Three months after insertion of the cement spacer, the patient presented to our clinic complaining of pain, instability, and swelling. Physical examination revealed a moderate effusion, with a painful arc of knee movement from 0° to 80°.

His medical history included uncontrolled diabetes, chronic obstructive pulmonary disease (COPD), and hypertension. He was a heavy smoker (25 packs/year) and had sustained two (2) previous myocardial infarctions, the last event being about six (6) months prior to attending our clinic. His Charlson Comorbidity Index (CCI) was 5 with an American Society of Anesthesiologists (ASA) score of 3.

The chance of a successful revision procedure was thought to be low, and salvage options of knee fusion or an above knee amputation (AKA) were discussed with the patient.

He refused amputation and we agreed to proceed with an arthrodesis. His preoperative investigations, C-reactive protein levels (CRP), and erythrocyte sedimentation rate (ESR) were 8 mg/L (range 1-10) and 20 mm/hr (range 0-22), respectively, and joint aspirate revealed mildly turbid fluid. No cell counts were available at our institution, and standard microbial cultures revealed no growth after five (5) days.

## 3. Technique

The patient was placed in the supine position with a sandbag under his right buttock, and fluoroscopy used to confirm an unobscured view of the hip, knee, and ankle joint. The operative field was cleaned and draped, and a midline incision made through the previous scar. The joint was exposed via a medial arthrotomy, and the cement spacer removed taking care to preserve the underlying bone. A complete synovectomy and debridement was performed, and the exposed bone ends fashioned using an oscillating saw. This was done to ensure minimum bone loss and maximum surface contact when apposed.

A second incision was made over the greater trochanter, and a bone awl used to establish a piriformis fossa entry point that allowed passage of a guide rod. The femoral shaft was then sequentially reamed until cortical chatter was heard. The guide rod was removed and passed via the knee joint into the tibial shaft which was reamed as described earlier. The guide rods were used to estimate the length of the femoral and tibial shafts, and this was extrapolated to calculate the length of the nails required to stabilize the entire lower limb.

Two femoral nails were selected, and a thick Rush rod inserted between the distal end of one nail and the proximal extent of the other. The Rush rod was prebent to establish three-point fixation providing a degree of stability at the junction while allowing for flexion at the fusion site.

**Figure 2:**
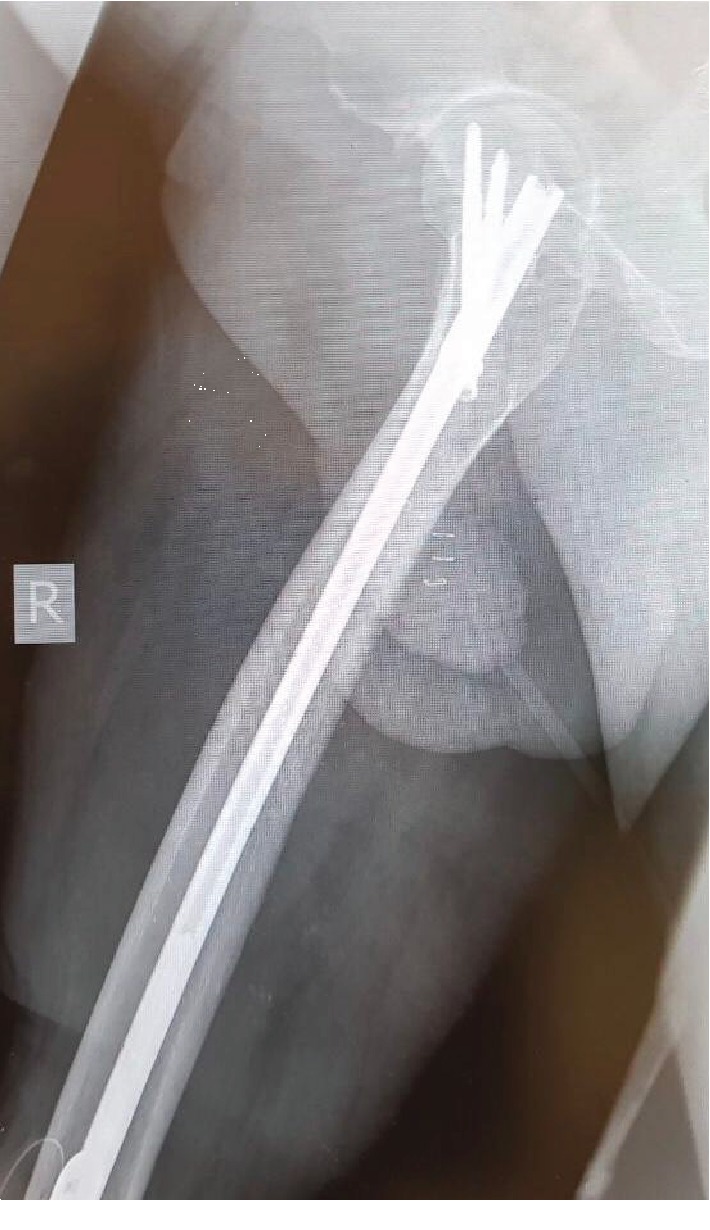
Radiograph shows femur with proximal locking in lateral view.

**Figure 3:**
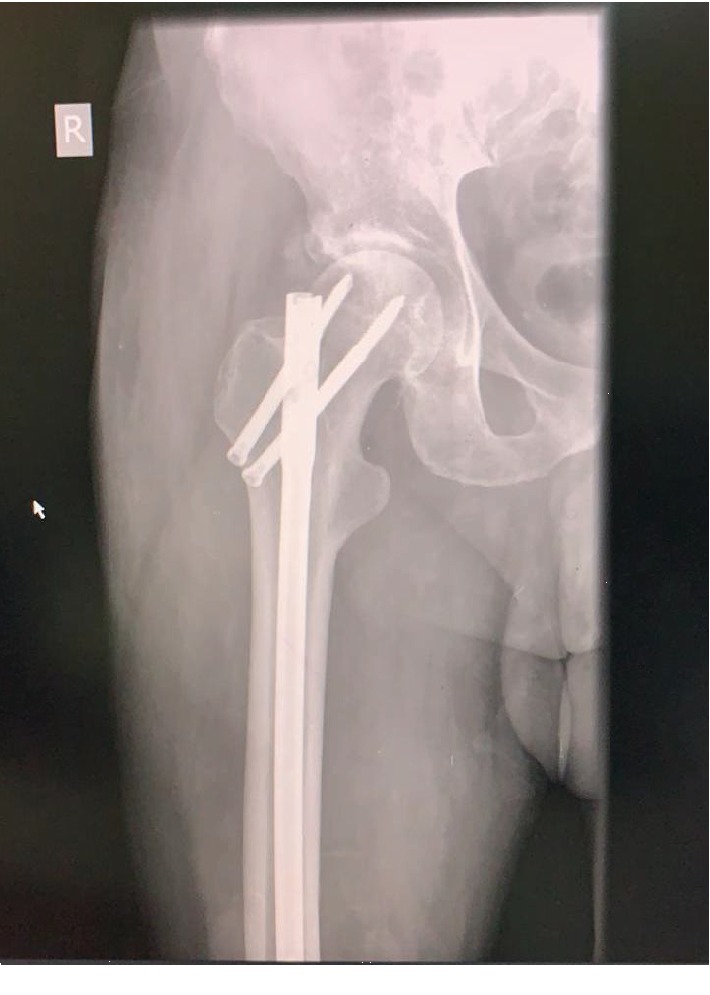
Radiograph shows femur with proximal locking in AP view.

The entire construct was introduced proximally via the piriformis fossa and guided through the knee and into the tibia. Care was taken to ensure that maximum bone apposition was achieved with good compression. The prominent anterior tibia was osteotomized and used for bone grafting. Locking was accomplished with the femoral jig proximally and distally using a free hand technique Figures 2 and 3.

**Figure 4:**
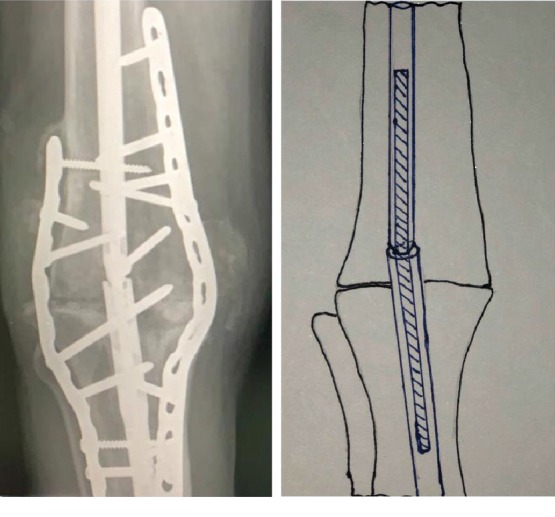
AP radiograph and illustration showing coupled nail with a Rush rod in situ.

**Figure 5:**
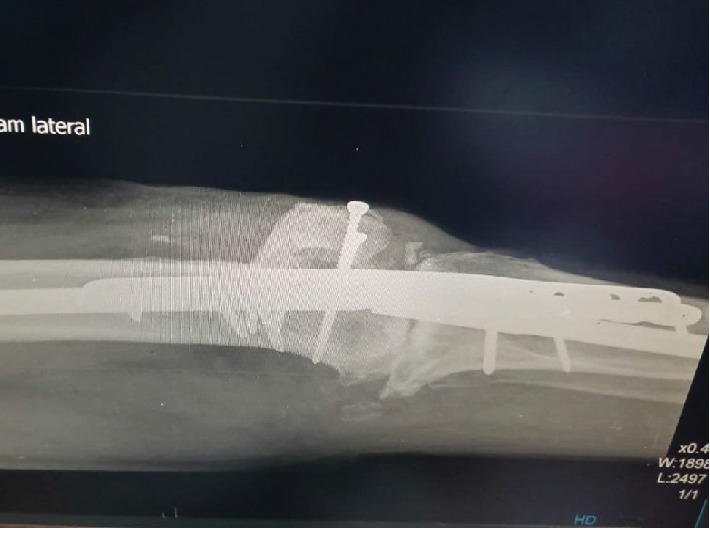
Radiograph shows knee fusion in lateral view.

Applying gentle varus-valgus pressure revealed movement at the osteotomy site. Two 3.5 mm DC (dynamic compression) locking plates were contoured and applied to the medial and lateral aspects of the knee. A combination of locking and nonlocking screws was used to ensure both stability and tight apposition of the plates to bone, facilitating soft tissue closure. The knee was retested, and no movement was noted at the fusion site thus confirming absolute stability. Hemostasis was secured, and the wound closed in layers Figures 4 and 5.

The patient received a one-week course of intravenous antibiotics and allowed immediate partial weight bearing. At two-month postsurgery, there was evidence of bridging trabeculae at the fusion, and he was permitted to fully weight bear.

**Figure 6:**
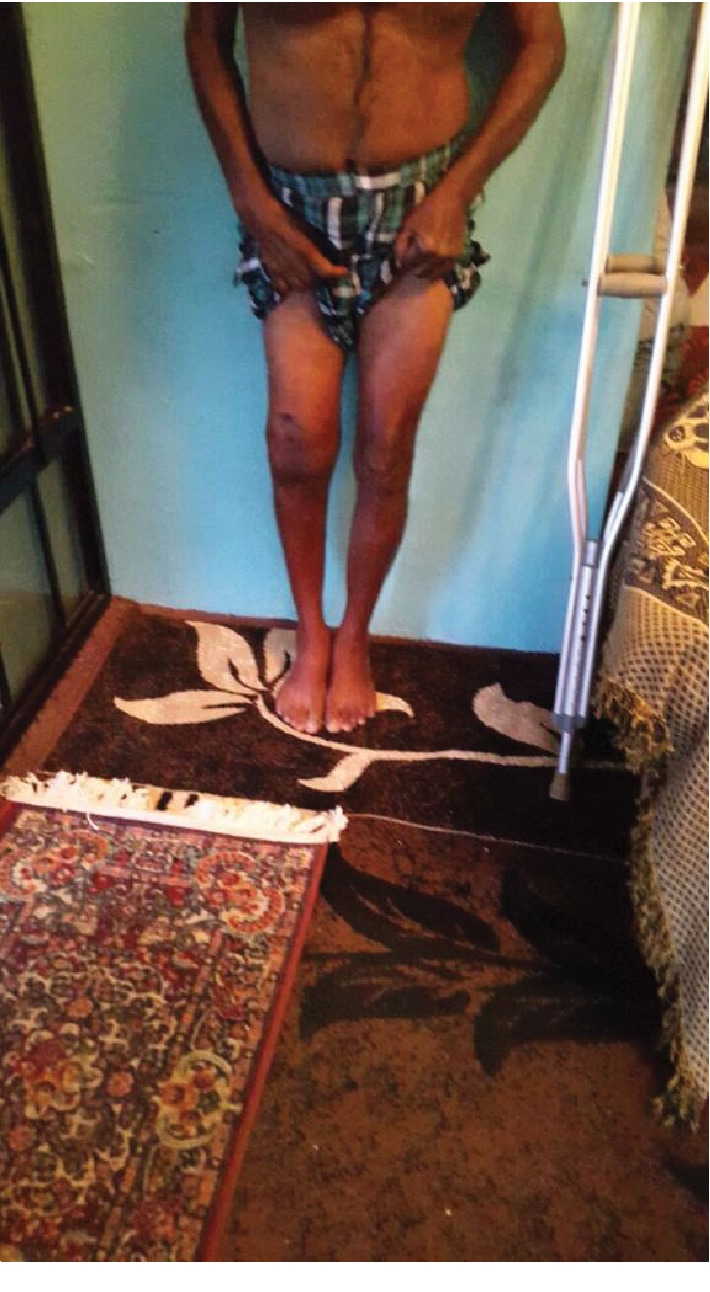
Clinical photograph of patient standing in AP view.

**Figure 7:**
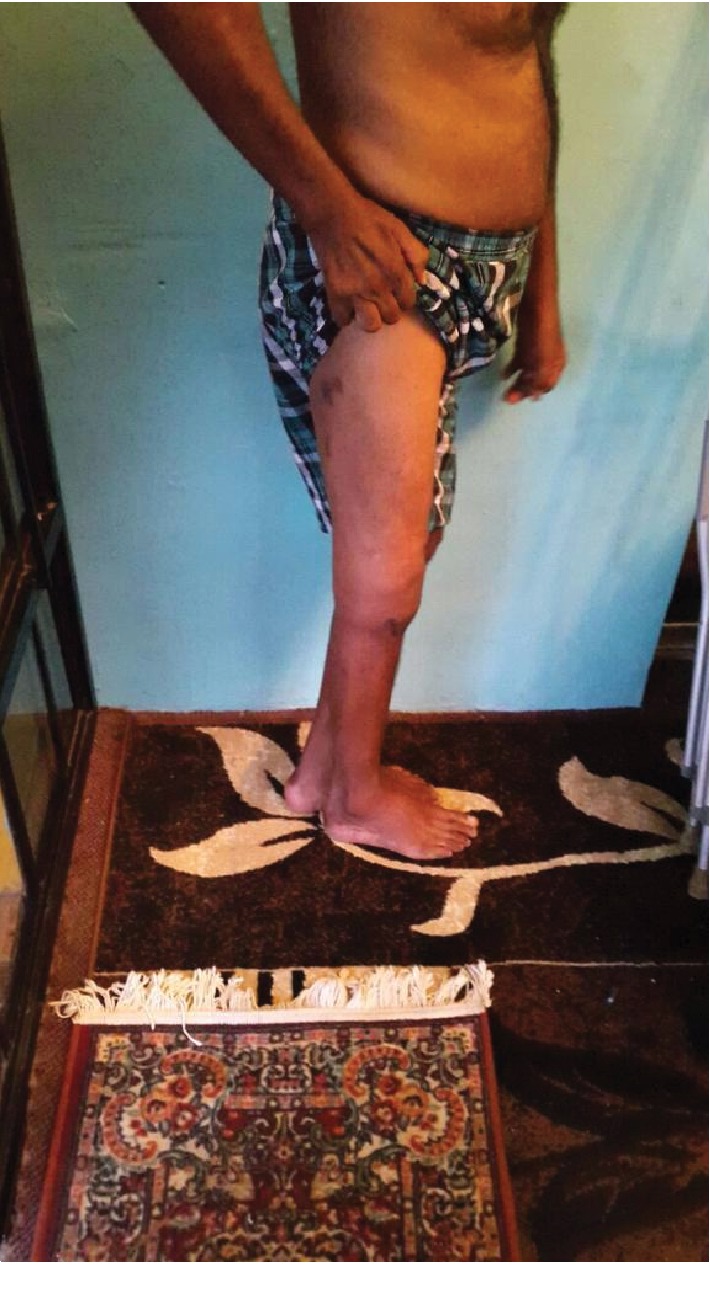
Clinical photograph of patient standing in lateral view.

One year from surgery, the patient is able to walk with no pain and can independently perform all activities of daily living (ADL) but requires a cane for balance. His right lower limb is 2.1 cm short requiring a shoe raise on the opposite side Figures 6 and 7. His Oxford Knee Score improved from 9 preoperatively to 31 at final follow-up.

## 4. Discussion

The most common indication for revision of a TKA is infection, followed closely by aseptic loosening [7]. An almost 50% increase in the revision burden of TKAs due to periprosthetic joint infections highlights the problem [8, 9]. This is compounded by the increasing costs of multiple revision procedures [10]. Despite excellent success with two-stage revision arthroplasty for the eradication of infection [3], on occasion, salvage procedures must be utilized. Fagotti et al. have highlighted the risk factors which can lead to failure of a two-stage revision TKR [11]. The patient had several of these poor prognosticators and would not have been suitable for revision TKR.

The salvage options available include resection arthroplasty, above knee amputation, and arthrodesis. The functional outcome of any salvage procedure is of obvious importance and plays a major role in the shared decision-making model.

Resection arthroplasty normally reserved for the nonambulant patient, for whom the removal of an infected draining sinus over the knee is a major benefit to their quality of life, would not have been acceptable to this patient.

The functional outcomes of above knee amputation (AKA) versus knee fusion have been assessed in a study by Hungerer et al. [12] They reported similar SF-12 (physical component) scores for both sets of patients but noted significantly better functional outcomes in AKA patients who were suitable for fitting of a microprocessor knee prosthesis. In our society, there is significant social stigma associated with amputees, and the prosthetics available are early generation which require considerable physical effort to permit ambulation. The patient's poor general medical health would have made it unlikely that he would have ever walked again even if he was fortunate enough to have been fitted with a prosthetic limb. For many of the forestated reasons, our patient refused amputation.

The patient decided to proceed with an arthrodesis and gave informed consent for his case to be reported in the literature.

The rates of knee arthrodesis for infected TKRs are decreasing worldwide as surgeons and institutions develop greater expertise in dealing with these challenging cases [13]. Improvements in technology with the development of tantalum and metaphyseal cones now allows surgeons to revise joints with massive defects [14]. In addition, improved reconstructive techniques and greater cooperation with the reconstructive plastic surgeons have reduced the risk of soft tissue breakdown.

Knee arthrodesis has been reported to have similar clinical outcomes to a two-stage revision TKR for infection, with the added benefit that patients are less likely to require other surgical procedures [15].

Knee fusion can be achieved either using a one- or two-stage procedure [16]. In the presence of chronic infection, some authors have demonstrated good results using a one-stage process to achieve knee fusion; however, caution is advised in patients who have had multiple previous attempts at revision, comorbidities, and infections with resistant organisms [17]. Much more commonly infected joints are treated by a two-stage arthrodesis.

In the infected case, it is recommended that the procedure be done in two stages, using preoperative aspiration, CRP, and ESR tests to confirm eradication of the infection before definitive fusion [3, 4, 16, 18–20]. Bargiotas et al. demonstrated that when a two-stage technique was used, and there was biochemical evidence that the infection was eradicated, excellent results could be attained [21]. In their study, solid union was achieved in ten of the twelve knees, with one patient having to undergo an above knee amputation due to recurrence of the infection and one case of nail breakage [21]. Fusion was achieved in our patient using a two-stage process, although the first stage was performed by his previous surgical team. Before proceeding to the second stage, all inflammatory markers returned to normal and the preop aspirate revealed no growth.

There are numerous techniques to achieve knee fusion, and traditionally, external fixation has been recommended as the method of choice in cases of sepsis [22]. However, several drawbacks limit its usage including prolonged application, bulkiness of device, and pin tract infections. Dual compression plates have also demonstrated very satisfactory outcomes for knee arthrodesis following failure of a knee arthroplasty; however, complications include femoral stress fractures and malalignment and the risk of persistent infection which limits its applicability [23]. More recently, intramedullary nailing has gained popularity amongst surgeons although there remain concerns with its use in cases of infection [24].

Several authors have reported excellent results with intramedullary nailing using a variety of devices with fusion rates of 83-100% at an average of 5.8 months (range 5.2–7) although reoperation rates of 10-17% have tempered enthusiasm for the technique [19, 25–29].

Benefits include rigid load-sharing allowing immediate weight-bearing, limited surgical exposure, and soft tissue dissection [30]. Additionally, the technique of nailing is one that is very familiar to surgeons reducing the need to learn any new steps [31]. For the many reasons given previously, we felt that fusion using an intramedullary nail would be most appropriate for our patient, but we faced additional challenges.

Arthrodesis with an intramedullary fusion nail can be accomplished with either a single or modular implant, the latter may allow minimally invasive access but its disadvantages include cost, lack of familiarity to the surgeon, and the use of additional equipment to ensure fit [29].

In our setting, the cost and availability of implants represent significant obstacles to timely treatment, frequently necessitating innovative thinking to affect a solution.

Stiehl and Hanel in 1992 reported eight cases, including two previously infected TKRs in which he used a single intramedullary nail and additional plate to achieve arthrodesis [32]. To the best of our knowledge, this is the first reported use of a nail-plate combination to achieve knee fusion. We modified Stiehl's technique to suit our specific case, utilizing two coupled intramedullary nails combined with bilateral plate fixation.

With the unavailability of a single long nail, two femoral nails were coupled with a Rush rod to give the desired length. Additional stability was achieved by contouring two locking plates on either side of the knee joint. While Stiehl et al. may have used one 4.5 mm dynamic compression plate, the newer 3.5 mm locking compression plates are less bulky, allowing the use of both locking and nonlocking screws, the former providing good fixation in the poor-quality bone and the latter allowing close apposition of the plate to the bone. Nail breakage, nonunion, and persistent infection are some of the complications which may be caused by a lack of absolute stability. Some authors have noted the technical complexity of achieving absolute stability with intramedullary nails, and this has instigated the development of specially designed fusion nails [17, 28, 33]. Our intramedullary construct was not rigid, and although the compression plates improved stability, the additional metalwork may have increased the risk of reinfection. On balance, we felt that the use of bilateral plates was justified since absolute stability would itself be a factor in reducing infection, while decreasing the time to fusion.

From a technical point of view, it is important to note that in order to achieve both good stability as well as a high fusion rate, broad contact must be obtained between the resected bones. Preparation of the bone ends should be meticulous producing large surfaces of good bleeding bone [21, 33]. In our case, this was achieved with the judicious use of an oscillating saw ensuring that the minimum amount of bone was removed and eliminating the need for allograft.

The composite construct provided firm contact and rigid fixation and retained blood supply which are the necessary requirements for successful fusion. We believe that the construct rigidity reduced the chance of nonunion, implant breakage, and persistence of infection which are common complications. Our method employs sound surgical principles and readily available low-cost implants combined with surgical techniques which are familiar to the surgeon, requiring no special training or additional equipment.

Postoperatively, the patient received seven days of intravenous antibiotics, although this is a relatively short course, we feel that this is satisfactory and represents good antibiotic stewardship. Successful treatment of infection is dependent primarily on a thorough soft tissue debridement and removal of all infected bone, reducing the bioburden [31]. In this case, our patient had two debridements with an intervening 3-month period of local antibiotic delivered via the cement spacer. Although some authors recommend long-term oral suppressive antibiotics, there is no compelling evidence to support this position and to date, our patient remains free of infection [34].

Some authors have stated that the treatment of a failed TKR should be conducted in centres with a special interest in knee arthroplasty [18]. Unfortunately, we do not have the luxury of such facilities locally. Often, it is a general orthopaedic surgeon who is faced with a failed TKR which requires revision or arthrodesis.

## 5. Summary

Knee fusion to salvage an infected total knee arthroplasty is a relatively uncommon surgical procedure. In a low-resource setting, the burden of treating the infected knee arthroplasty frequently falls on the general orthopaedic surgeon. We believe that the technique described is inexpensive and within the surgical skill set of most orthopaedic surgeons and should be one of the options to treat these challenging cases.
